# Hybrid Nano-GdF_3_ contrast media allows pre-clinical *in vivo* element-specific K-edge imaging and quantification

**DOI:** 10.1038/s41598-019-48641-z

**Published:** 2019-08-20

**Authors:** Niki Halttunen, Frederic Lerouge, Frederic Chaput, Marc Vandamme, Szilvia Karpati, Salim Si-Mohamed, Monica Sigovan, Loic Boussel, Emmanuel Chereul, Philippe Douek, Stephane Parola

**Affiliations:** 1 Laboratoire de Chimie, Université de Lyon, École Normale Supérieure de Lyon, Université Claude Bernard Lyon 1, CNRS UMR 5182, 46 allée d’Italie, 69364 Lyon, France; 2 VOXCAN, 1 avenue Bourgelat, 69280 Marcy l’Etoile, France; 3 CREATIS, CNRS UMR 5220, INSERM U1206, Université de Lyon, Lyon, France; 4 Radiology Department, Hospices Civils de Lyon, Lyon, France

**Keywords:** Preclinical research, Imaging techniques and agents

## Abstract

Computed tomography (CT) is a widely used imaging modality. Among the recent technical improvements to increase the range of detection for optimized diagnostic, new devices such as dual energy CT allow elemental discrimination but still remain limited to two energies. Spectral photon-counting CT (SPCCT) is an emerging X-ray imaging technology with a completely new multiple energy detection and high spatial resolution (200 μm). This unique technique allows detection and quantification of a given element thanks to an element-specific increase in X-ray absorption for an energy (K-band) depending on its atomic number. The main contrast media used hitherto are iodine-based compounds but the K-edge of iodine (33.2 keV) is out of the range of detection. Therefore, it is crucial to develop contrast media suitable for this advanced technology. Gadolinium, well known and used element for MRI, possess a K-edge (50.2 keV) well suited for the SPCCT modality. The use of nano-objects instead of molecular entities is pushed by the necessity of high local concentration. In this work, nano-GdF_3_ is validated on a clinical based prototype, to be used as efficient *in vivo* contrast media. Beside an extremely high stability, it presents long lasting time in the blood pool allowing perfusion imaging of small animals, without apparent toxicity.

## Introduction

Since its introduction over 40 years ago^1,2^, CT has become one of the most used imaging modalities with MRI for clinical purposes in hospitals, especially in the emergency room. CT has known many evolutions most of them aiming to improve the image resolution, the scanning speed or to lower the radiation dose received by the patient. These developments have led to the very recent dual energy CT currently available commercially. However, improvements to the CT technology are still ongoing and new improvements are being developed at the present day, among the most recent ones is the K-edge imaging. After the concept was initially proven at synchrotron facilities^3^, the system and the reconstruction algorithms have been developed. Today the technology is matured enough to build prototypes under the form of the spectral photon-counting CT^4–8^.

Spectral photon-counting CT (SPCCT) is an emerging X-ray imaging technology with a completely new type of detection chain which pairs high count-rate capabilities to multiple energy discrimination and high spatial resolution (200 μm)^9–14^. This energy discrimination allows, in comparison with standard CT technology, a better sampling of the spectral information from the transmitted spectrum. It gives additional physical information produced during matter interaction, including photo-electric, Compton and K-edge effect. The K-edge can be described as an element-specific imaging corresponding to the measurement of the increase of the mass attenuation coefficient, at an energy corresponding to the K-band of the element^12^. Furthermore, not only K-edge imaging allows to detect a specific element, it also enables to quantify it. It is thus possible to accurately locate and dose the contrast media within the tissues. Since the energy range of the X-ray tube emission in the SPCCT is 30 keV to 120 keV, only elements with a K-edge value between these values can be detected^13,15–18^. The main contrast agents used in CT scan today are iodine-based compounds; however, due to its low K-edge (33.2 keV), iodine is not suitable for K-edge imaging because of the photon starvation at that energy. Therefore, it is necessary to develop new systems suitable for this technology.

Gadolinium is an element with a k-edge of 50.2 keV, which is within the detection range of the X-ray tube emission. This element is already well known and used for its magnetic properties as contrast media for MRI under its Gd^3+^ cage-complex form (DOTAREM, GADOVIST) in clinics or nanoparticles in research developments^19,20^. Despite the current use of molecular systems on human patients, drawbacks have been recently pointed out with the leaking of gadolinium ions that can accumulate in the brain^21^. Such molecules also show very short lifetime in the body (fast renal clearance) associated with a relatively small amount of gadolinium in mass. In order to be used as contrast agents with SPCCT, Gadolinium based systems must (i) be highly stable, (ii) contain a high amount of element for a better imaging, and (iii) show a blood half-life long enough to allow perfusion imaging. Recent development reports the use of molecularly based contrast media^22^. However as previously pointed out, the requirements on such technology, in particular the local concentration and the stability towards Gd^3+^ release, led us to investigate the potential of hybrid nanoparticles with high gadolinium loading and stable inorganic core.

We discuss herein the preparation and *in vivo* evaluation of ultra-small GdF_3_ nanocrystals as stable efficient contrast media for angiography with SPCCT clinical prototype suitable for preclinical studies, with a biodistribution study on mice and K-edge imaging on rats. These nanoparticles present two main advantages compared to the organometallic gadolinium complexes. First, they feature a very high stability with no possible gadolinium leakage^23,24^. Secondly, these nanoparticles show a high payload of gadolinium ions (from 10^4^ to 10^6^ Gd^3+^ depending on the exact particle diameter, between 9 nm and 25 nm). These systems provide thus a very high local density of active ions, making them efficient probes for various imaging modalities.

## Results

### Particle synthesis and characterization

**Figure 1:**
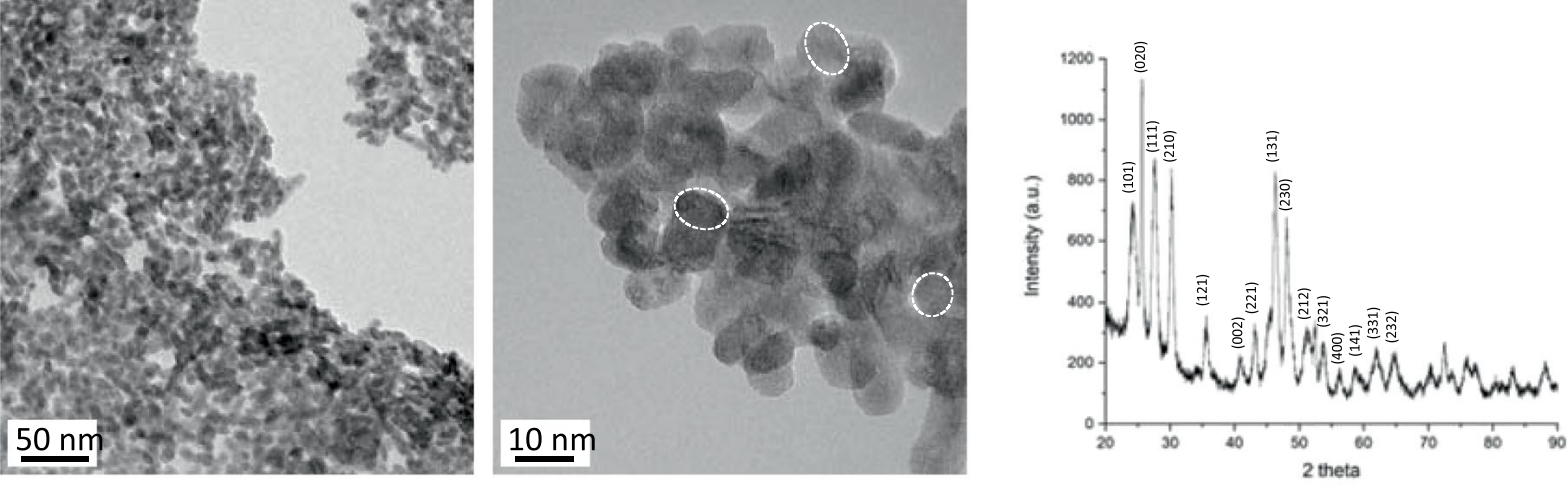
Transmission Electron Microscopy of GdF_3_ nanoparticles (left); High resolution of the crystalline nanoparticles (middle) and powder X-Ray Diffraction pattern (right).

The GdF_3_ nanoparticles were synthetized following a previously described process, with few modifications^23,24^. Briefly, a solution of gadolinium chloride in a mixture of ethylene glycol and 2-pyrolidinone was reacted under solvothermal conditions with a mixture of hydrofluoric acid (HF) in 2-pyrrolidinone. The obtained charge transfer complex acts both as a precursor of fluoride ions and a stabilizing agent preventing further growth of the nanoparticles. After several purification steps GdF_3_ nanoparticles gave a perfectly stable suspension in water. Transmission electron microscopy (TEM) and dynamic light scattering (DLS) showed that the nanocrystals exhibited an average size of about 10 nm with a low polydispersity and a hydrodynamic diameter centred at 14 nm. TEM images of the nanoparticles confirmed the crystalline (Fig. 1). The diffraction pattern of GdF_3_ (Fig. 1) was in good agreement with the TEM pictures revealing the presence of highly crystalline nanostructures with an orthorhombic phase.

Despite the fact that GdF_3_ nanoparticles showed great stability in water, surface modification needed to be considered for biological applications, specially to ensure a long remaining time in blood after *in vivo* injection. The surface of the GdF_3_ nanocrystals was thus functionalized using phosphonate-based Polyethyleneglycol ligands leading to both biocompatible and stable hybrid nanoparticles. Usually, anchoring groups used for surface functionalization of similar systems are carboxylic acids or their derivatives such as carboxylate salts, esters or amides. However, phosphonates groups were more suitable due to their strong efficiency to bind with the lanthanide at the surface of the particles. Effective surface modification of the nanoparticles was first observed by DLS, which showed an increase in the hydrodynamic diameter from 14 up to 18 nm. Fourier transform Infrared (FT-IR) analysis were also in agreement with the presence of ligands with specific signals of the phosphonate groups in the 950–1100 cm^−1^ range corresponding to the P-OH (950 cm^−1^) and P-O (1105 cm^−1^) vibrations and signals corresponding to the C-H stretching mode at 2870 cm^−1^.

### Radioopacity calibration

**Figure 2:**
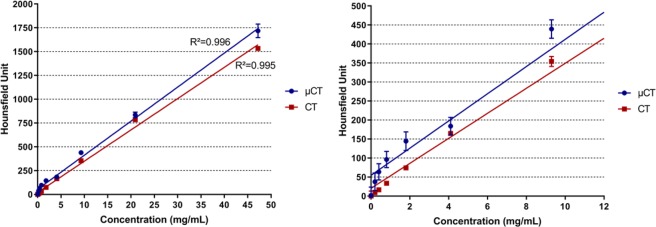
Calibration of GdF_3_, radio opacity measurements (HU) as a function of Gadolinium concentration. The right plot represents a zoom section of the 0–12 mg/mL range issued from the left plot.

To determine injection volume and product concentration for the *in vivo* application, a complete toxicology study is needed with dose escalation. In this first approach, the determination of the radio-opacity of the product was evaluated by CT and µCT imaging in tubes. To this end, the ability of the nanoparticules to absorb X-Ray was evaluated on suspension of GdF_3_ in NaCl 0.9% with various concentrations in Gd^3+^ (Fig. 2). A linear behavior was observed in both cases on a wide range of concentrations from 0.001 mg/mL up to 47 mg/mL (ie 0.01 mM up to 300 mM) with a constant increase of HU units (R² = 0.995, slope 4.88). Interesting results were observed in the lower range of concentrations. Considering HU value in muscle and soft tissue, a minimum value of 100 HU was needed in order to have a sufficient contrast, therefore concentrations of at least 2.35 mg/mL were necessary in order to observe the particles in biological tissues. This result proved the efficiency of the nanoparticles to act as contrast agents with CT imaging.

### *In vivo* biodistribution

*In vivo* studies were conducted on mice in order to evaluate the behaviour of the nanoparticles *in vivo* and confirm their efficacy as blood pool contrast media. Before injection, the particles were dispersed in physiological serum (NaCl 0.1 M in water) with various concentrations of gadolinium atoms, ranging from 20 mM to 300 mM. Once the particles were in solution, the mixture was stable for several months with no noticeable change.

**Figure 3:**
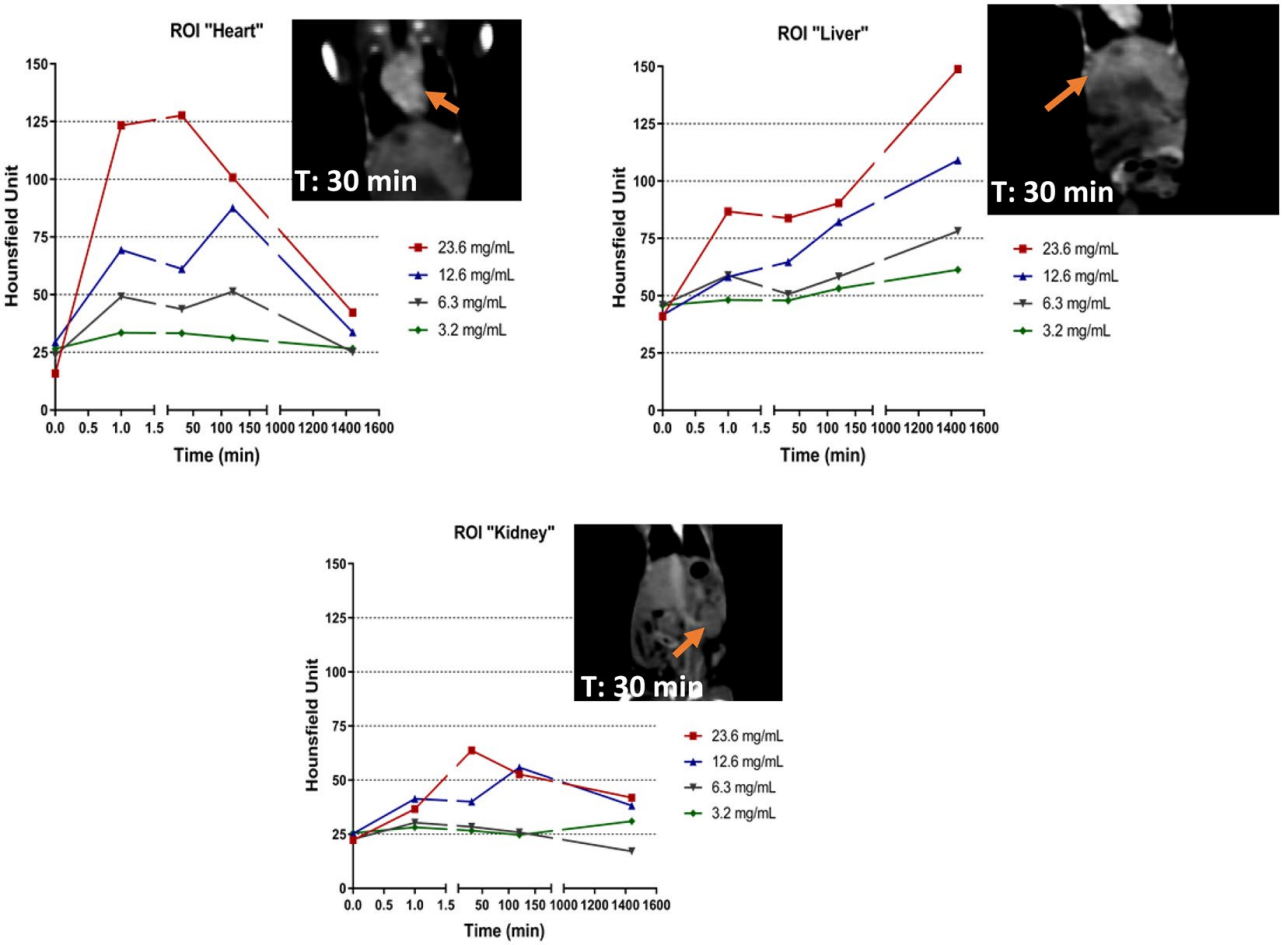
*In vivo* monitoring and CT images of the hybrid nano-GdF_3_ particles in the Heart (top left), the liver (top right), the kidney (bottom). CT images at T = 0 and 1 minute are shown in Fig. 4.

**Figure 4:**
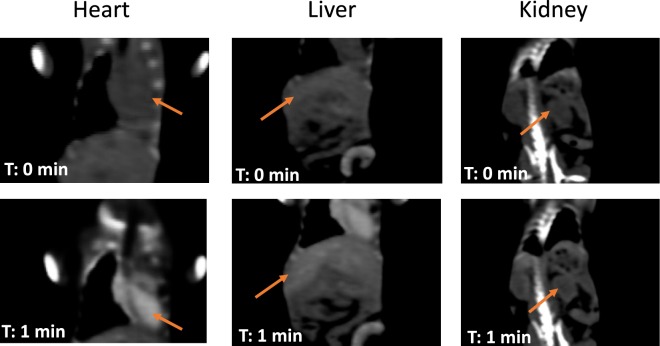
CT images at T = 0 and 1 minute.

Biodistribution was performed *in vivo* by monitoring the heart, liver and kidneys on a series of 4 animals with 5 different concentrations (20 mM, 40 mM, 80 mM, 150 mM and 300 mM) of Gadolinium, few minutes and up to 24 hours after injection (Figs 3 and 4). No animal mortality was observed during the study. Intense signal was noticeable in the heart of the animals after injection with a maximum at 30 min. Whatever the gadolinium concentration was, the strong contrast faded slowly after more than two hours, proving the long remaining time of the nanoparticles in the blood pool. These observations were confirmed in the case of the liver and a slow increase of the contrast was measured with a maximum corresponding to 24 hours after the injection. Such result demonstrated the slow uptake of the particles by the reticuloendothelial system and confirmed their high stability in the blood. These observations were completed with a monitoring of the kidney, where no contrast *ie* no localization of the particles was observed in that area. This also confirmed the fact that the majority of the particles were located in the liver after one day of experiment, without renal clearance.

The hybrid nano-GdF_3_ contrast media is thus an efficient tool for CT imaging, providing great contrast with a long-lasting period of time in the blood circulation. It can further be used as efficient perfusion imaging contrast media.

### *In vivo* K-edge imaging

**Figure 5:**
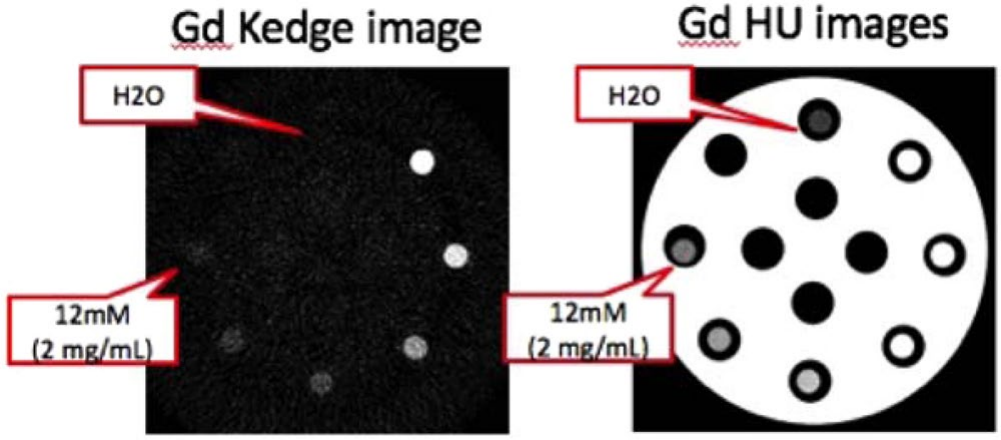
(Left) SPCCT gadolinium K-edge image of a phantom consisting of GdF_3_ suspensions of various gadolinium concentrations from 2 mg/mL up to 20 mg/mL (12 mM to 120 mM respectively) (Right) Same phantom observed with SPCCT conventional image.

Evaluation of the k-edge imaging with the hybrid nano-GdF_3_ contrast media was performed using SPCCT prototype (Philips Healthcare, Haifa, Israel), first *in vitro* on phantom tubes with a range of gadolinium concentrations (2–20 mg/mL). Comparison with HU image was also conducted on the same apparatus (Fig. 5). K-edge observations only showed signals due to the element gadolinium in the phantom and the tube holder did not appear in the image. This result showed the specificity of the imaging technology for one element. In the case of HU observations corresponding to a classical scanner CT, both tubes and sample holder were imaged. Results were in good agreement with previous measurement and the limit of detection was observed for a concentration of 2 mg/mL of gadolinium.

**Figure 6:**
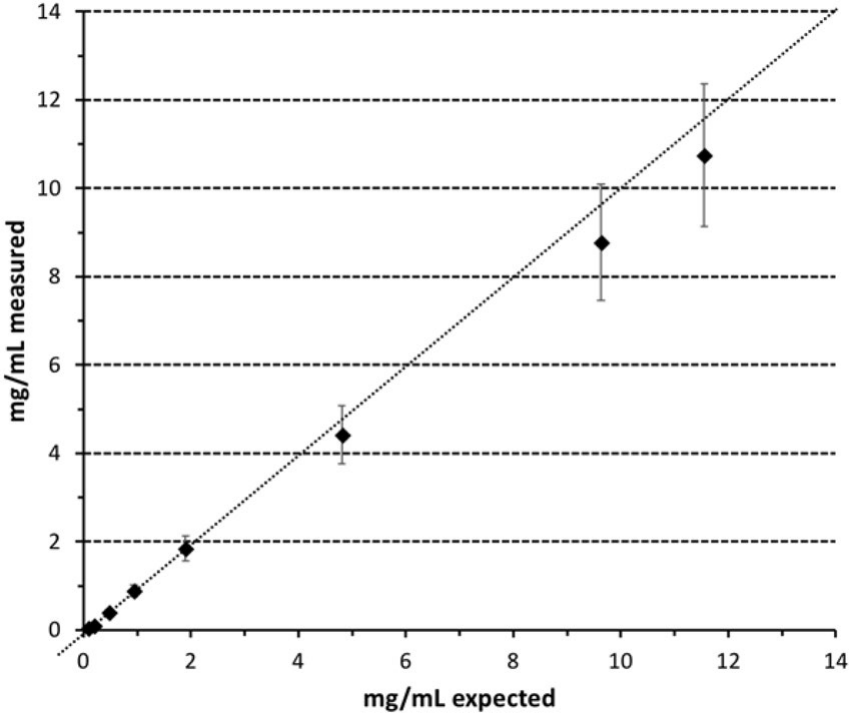
Correlation between measured concentration of gadolinium with K-edge and expected concentrations of the corresponding solutions determined by ICP.

Since this modality allows the quantification of a given element, a correlation between expected and measured concentrations was performed to assess and validate the accuracy of the method for gadolinium. A series of nanoparticles suspension in water at various concentration of gadolinium was first evaluated with ICP. The solutions were then analyzed by k-edge and the results were compared (Fig. 6).

The obtained curve shows a good correlation between the concentration obtained from ICP and the measured ones with the spectral scanner technology in the lower range (0–4 mg/mL). A slight deviation is observed above 4 mg/mL and the measured concentration is always below the expected result but still in the error margin.

**Figure 7:**
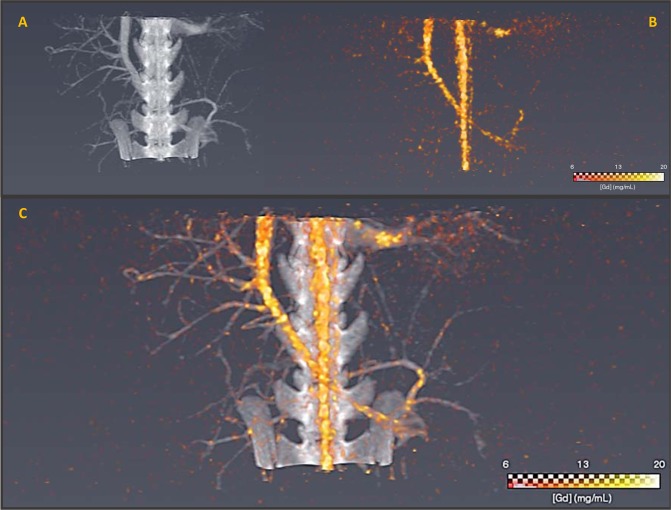
SPCCT conventional HU image (**A**) SPCCT gadolinium K-edge image (**B**) and overlay between SPCCT conventional HU and Gd K-edge images (**C**) of abdomen of rat after GdF_3_ injection (5 min). Both images are reconstructed on an isotropic voxel grid at 250 µm * 250 µm * 250 µm.

Evaluation of GdF_3_ contrast media was performed after systemic injection *in vivo* on healthy rat, for perfusion imaging of the abdomen. Acquisitions were performed before and after the injection of 2.5 mL of 1 M GdF_3_ (gadolinium amount: 10 mg/g of animal) by focusing on the arteriovenous system (Fig. 7).

First, when imaging was performed through conventional CT technology, a strong HU signal was visible (grey) allowing the visualization of vascularization (small vessels and arteries). The observation of the blood network agreed with the long persistence of the GdF_3_ in the blood stream. K-edge imaging (in yellow) provided a specific image of the contrast media in the animal vasculature. The signal represented specifically the agent of interest in the arteries and the surrounding vessels, although the image was noisy such as it has been related in a previously published paper^13^ where the authors found a noise value within the gadolinium images around 0.5 mg/mL. It was thus possible to distinguish the profile of the blood vessel thanks to the Gadolinium element of the particles and no bones (spine or hips) were observed. Merging of both imaging modalities allowed the observation of the abdomen vascularization of the animal and the localization of the particles in the blood stream. Such results are very encouraging for further studies in the frame of vascularization-associated pathologies such as ischemia or cancer angiogenesis.

### *In vivo* quantification

**Figure 8:**
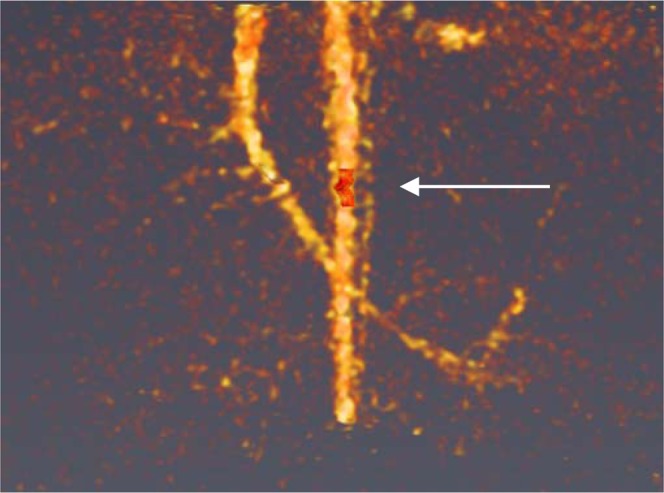
Delimited box (in red) for Gd^3+^ quantification in the aorta.

With conventional CT imaging, the characterization of tissue or amount of contrast media relies only on the signal attenuation. This lack of specific and quantitative evaluation of contrast agent distribution has been overcome using the SPCCT k-edge modality. The specific imaging of gadolinium element in the new probe became possible and the quantification was performed using ROI manually delineated in aorta (Fig. 8).

Thus, a gadolinium concentration of 17.3 mg/mL was directly quantified whereas when using a conventional CT, a real concentration determination was not possible. Since the modality is directly related to the atomic number of the element and given the fact that Gadolinium’s is much higher than surrounding matrix such as water or plasma, the correlation between ICP and measured concentration (Fig. 6) applies.

This result shows the potential of SPCCT to determine a gadolinium concentration in a given volume. Using this technology and the scanning rapidity, it is possible to track the bolus injection of contrast media and to quantify tissue perfusion such as in the case of myocardial infarction.

## Conclusions

Spectral photon-counting CT (SPCCT) imaging shows today an extremely high potentiality for medical diagnostic and requires simultaneous combination with efficient contrast media with compatible K-edge and high local element density. Nano-GdF_3_ appears to be a good candidate for use as contrast media in SPCCT. It presents a long blood half-life allowing *in vivo* injections and imaging of small and medium animals models. It shows extremely high stability with strong potential in term of non-toxicity. Not only imaging on the contrast media can be performed *in vivo*, but element specific quantification is possible in region of interest and as performed during imaging acquisition. The results on the hybrid nano-GdF_3_ as long-lasting blood pool agent are extremely encouraging, allowing future perfusion imaging at preclinical stage.

## Methods

### Animal experiments

All experiments were performed in accordance with relevant guidelines and regulations. The animal experiments were carried out according to the principles of laboratory animal care and European legislation (Council Directive No. 2010/63/UE on the protection of animals used for scientific purpose). This protocol was submitted to our local ethical committee (C2EA-18 VETAGRO-SUP) and to the French authorities and received a favorable opinion on 09/11/2014 (Project No. 1427_v3).

Female Balb/cJRj mice and male Wistar Rats (RjHan:WI) were purchased from JANVIER Laboratories (7 weeks old and 6 weeks old respectively) weighing about 25–30 g for the mice and 180–220 g for the rats were used for the *in vivo* studies. Mice and rats were provided with standard mouse food and water ad libitum and maintained under conventional housing conditions in a temperature-controlled room with 12-hour dark–light cycle.

For pharmacokinetics and biodistribution studies: mice were divided into five groups of treatment which received different nanoparticles concentration: 20 mM, 40 mM, 80 mM, 150 mM and 300 mM.

### Imaging protocol

CT acquisitions were performed using two CT systems. The first one, for biodistribution study, is a standard CT (General Electric BrightSpeed 16 Elite, GE, France) using 120 kV, 150 mA, a field-of-view (FOV) of 50 cm and a spatial resolution of 310 µm at consecutive time points (T = 0, 1, 30, 120 minutes and 24 hours).

The second one, for Gd^3^^+^ K-edge imaging, is a spectral photon-counting prototype CT system (SPCCT, Philips Healthcare, Haifa, Israel). It is a modified base clinical system with a conventional scintillator detector replaced by a photon-counting detector of smaller coverage. It is a equipped with a conventional X-ray tube, and a conventional small bowtie filter, delivering a radiation dose similar to a standard CT. For information, a CTDIweighted at 0.74 mGy has been measured for an axial scan over 360 degrees at 100 mA tube current and 120 kVp tube voltage. The scan FOV was 168 mm in-plane, with a z-coverage of 2.5 mm in the scanner iso-center. Axial and helical scans over 360 degrees were performed at 100 mA tube current and 120 kVp tube voltage with a scanner rotation time of 1 s and 2400 projections per rotation.

Images of gadolinium were generated via the K-edge technique taking benefit from the energy resolving detectors. This technique is based on the detection of the K-edge effect of a material defined as the binding energy of its K-shell electron. To allow this, the SPCCT is based on hybrid photon counting detectors, ChromAIX2 application-specific integrated circuits combined with cadmium zinc telluride (CZT) as sensor material, and operates in single photon-counting mode with energy discrimination^25^. The photon counting detectors allowed up to five consecutive energy bins between 30 and 120 keV. 5 thresholds were adjusted in order to allow photon energy–based discrimination of the gadolinium. 2 thresholds just below and above their K-edges, at 50.2 keV, are needed such as demonstrated in previous papers^26–28^. One additional energy threshold served as a noise threshold and was set to 30 keV. Hence, the energy thresholds were set at 30, 51, 64, 72, 85 keV for the gadolinium study.

For each pixel, a maximum likelihood estimator was used to derive an equivalent water-thickness per pixel from the photon counts in the five energy bins. Sinograms were individually reconstructed with a reconstruction algorithm on a 0.25 × 0.25 × 0.25 mm^3^ voxel grid. Conventional images from the SPCCT were reconstructed from the water-thickness equivalent sinograms using a filtered back-projection algorithm. Gadolinium images were reconstructed from the five multi-energy sinograms after a discrimination process between 3 materials-based in the purpose to provide a three-material water-iodine-gadolinium basis. This process is based on a forward projection model (per detector) and maximum likelihood to determine the energy-dependant attenuation of the gadolinium^28^.

Regions of interest were manually drawn using a dedicated Software (Avizo, ThermoFischer) within the heart, liver and kidney (renal cortex) for quantification of the attenuation values in Hounsfield units on the SPCCT conventional HU images, and the concentrations in mg/ml on the SPCCT K-edge images.
